# The applications, complications, and management of neonatal peripherally inserted central catheters (n-PICCs): a narrative review

**DOI:** 10.3389/fped.2026.1902619

**Published:** 2026-08-05

**Authors:** Lei Yang, Qiaoling Fan, Lingyan Fan, Jiayu Qiu

**Affiliations:** 1 Department of Orthopedics, Shanghai Children’s Hospital, School of Medicine, Shanghai Jiao Tong University, Shanghai, China; 2 Department of Neonatology, Shanghai Children’s Hospital, School of Medicine, Shanghai Jiao Tong University, Shanghai, China

**Keywords:** complications, intervention, neonate, nursing, peripherally inserted central catheters, specialized team

## Abstract

The neonatal peripherally inserted central catheters (n-PICCs) is a commonly used vascular access device in neonatal intensive care, yet its associated complications significantly affect patient outcomes. This narrative review aims to summarize current status of n-PICCs, major complications and risk factors, and evidence-based preventive strategies. A literature search was conducted in PubMed, Web of Science, and Embase for articles published between January 2000 and February 2026, and relevant studies were synthesized and discussed. The findings demonstrate that the incidence of n-PICCs-related complications ranges from 10% to 40%, encompassing catheter-related bloodstream infection, thrombosis, cardiac tamponade, and mechanical failure. Key risk factors include low birth weight, small gestational age, prolonged catheter dwell time, malposition of the catheter tip, and improper insertion site selection. In terms of preventive strategies, interventions such as ultrasound guidance, intracavitary electrocardiography, antimicrobial-impregnated catheters, tissue adhesive securement, and bundle care strategies have been associated with reductions in specific complications in observational and quasi-experimental studies, although the evidence quality varies, while specialized vascular access teams and standardized training serve as important foundations for ensuring procedural quality and patient safety. Based on these findings, this review proposes that neonatal vascular access management should shift from single-intervention approaches toward system-based frameworks. Integrating technical optimization, material selection, standardized procedures, and organizational management into a comprehensive safety system covering the full process-assessment, insertion, maintenance, removal, and monitoring-represents a key pathway for reducing complications and ensuring patient safety. Future research should focus on multicenter randomized controlled trials, improving risk prediction models, and advancing the clinical application of intelligent monitoring technologies and evidence-based practice dissemination.

## 1 Introduction

In neonates, particularly those born preterm or with very low birth weight, the gastrointestinal tract is often immature, necessitating prolonged parenteral nutrition support (1). Additionally, critically ill neonates frequently require continuous infusion of hyperosmolar or irritant medications, such as vasoactive agents and potent antibiotics (2). Establishing safe and reliable venous access is therefore essential to ensure effective treatment. Recently, an international position statement has proposed the NAVIGATE nomenclature for vascular access devices, recommending the term n-PICC for neonatal peripherally inserted central catheters (n-PICCs) to standardize terminology across research and clinical practice (3). Since its first use in neonates in the 1970s, the n-PICCs have become one of the most commonly used central venous access devices in neonatal intensive care units (4). This is largely attributed to its ease of bedside insertion, extended dwell time (weeks to months), and lower complication profile compared with surgically placed central lines (5). It is estimated that approximately 2.5 million patients in the United States receive a PICC annually, with particularly widespread use in the NICU (6). The use of PICC significantly reduces the pain associated with repeated peripheral venipuncture, preserves peripheral vasculature. It also provides a secure route for the administration of hyperosmolar nutritional solutions and irritant medications.

However, the increasing use of PICC has been accompanied by a growing concern over device-related complications. Reported incidence rates of n-PICCs-related complications range from 10% to 40%, encompassing catheter-related bloodstream infection, thrombosis, cardiac tamponade, and various mechanical issues such as catheter occlusion, phlebitis, damage, and dislodgement (7–9). These complications can lead to unplanned catheter removal, prolonged hospitalization, increased healthcare costs, and in severe cases, life-threatening events (7, 8). Identifying risk factors and implementing targeted preventive strategies has therefore become a critical focus in n-PICCs management.

Recent advances in medical technology and the growing emphasis on evidence-based practice have led to the development of several interventions. These interventions are aimed at preventing n-PICCs-related complications. Ultrasound guidance improves first-attempt success rates and placement accuracy, thereby reducing vascular injury (10, 11). Intracavitary electrocardiography enables real-time tip positioning, minimizing radiation exposure and reducing malposition-related events (12, 13). Antimicrobial-impregnated catheters offer a potential strategy for reducing bloodstream infections, though their routine use in neonates remains debated (14, 15). Tissue adhesive securement has been shown to reduce catheter dislodgement and migration (16). Bundle care strategies, including integrating hand hygiene, maximal sterile barriers, chlorhexidine skin disinfection, and daily assessment of catheter necessity. These strategies have demonstrated significant reductions in infection rates and extended catheter dwell time (17, 18). Moreover, the establishment of specialized PICC teams and standardized training programs has been recognized as essential for improving procedural success and patient safety (19, 20).

**Figure 1:**
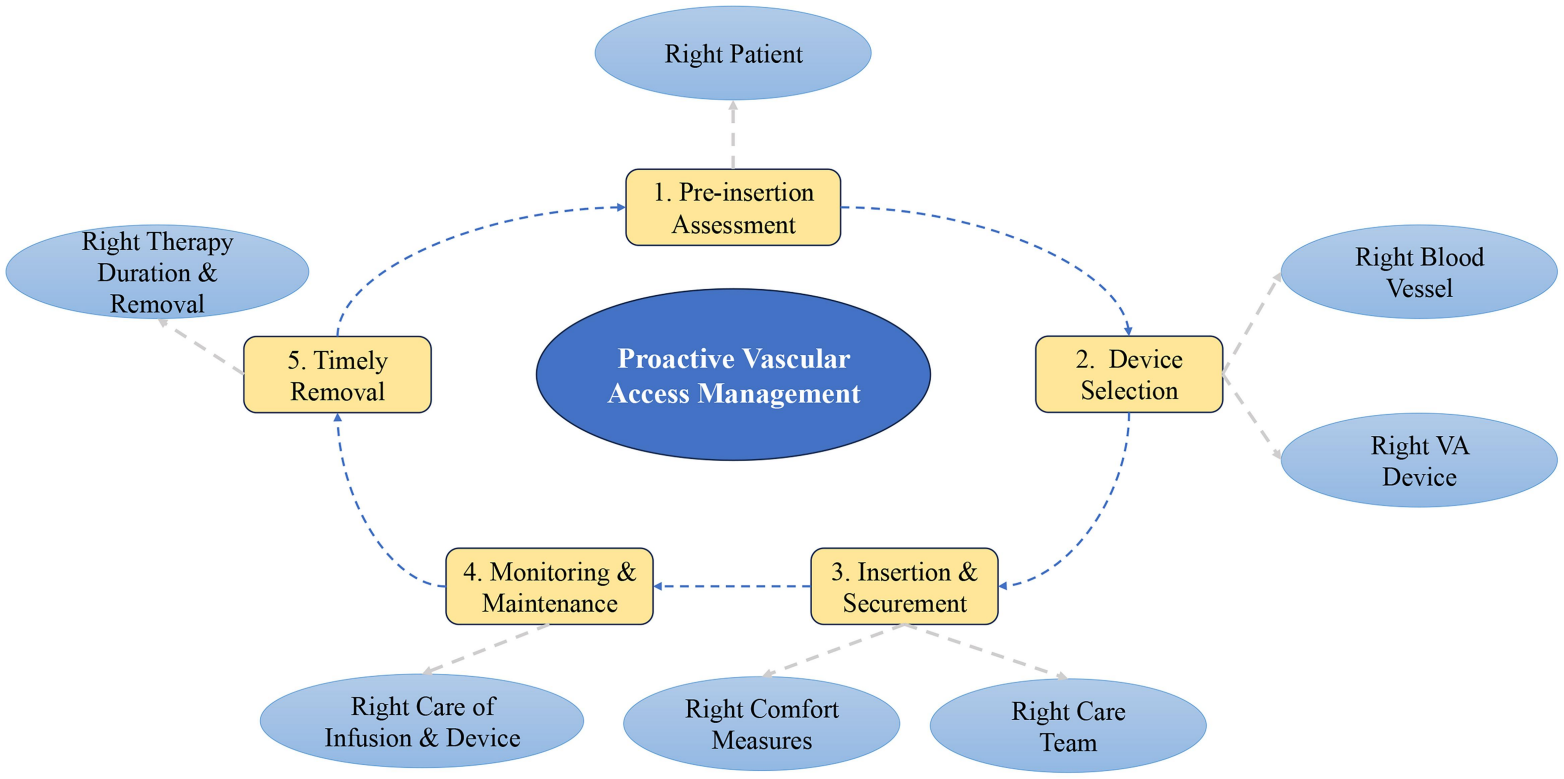
Neonatal vascular access safety framework based on the 7-rights model [Adapted from van Rens MFPT et al. (21)].

Despite the promising results of these individual interventions, most studies have focused on isolated aspects of PICC care. There remains a lack of comprehensive, system-level frameworks that integrate the full continuum of vascular access management-from assessment and insertion to maintenance, removal, and monitoring. Recently, van Rens et al. (21) proposed the “7-Rights Framework for Neonatal Vascular Access,” which emerged through international expert consensus. This framework uses the concept of patient rights, encompassing the Right Patient, Right Care Team, Right Comfort Measures, Right Vascular Access Device, Right Blood Vessel, Right Care of the Infusion and Device, and Right Therapy Duration and Device Removal. It integrates best evidence-based practice, ethical considerations, and family involvement into a structured, patient-centered model that guides the entire vascular access process—from planning and device selection to insertion, maintenance, monitoring, and quality control. A Vascular Access Management Plan based on this framework has the potential to individualize care, improve consistency, enhance patient safety, and facilitate quality improvement initiatives. The conceptual structure of this framework and its integration with the full clinical workflow is illustrated in Figure 1.

Accordingly, this review adopts the 7-Rights Framework as its conceptual foundation, organizing the evidence on PICC-related complications and preventive strategies according to the key domains of this framework—patient assessment (Right Patient), procedural technique (Right Blood Vessel, Right VA Device), monitoring and maintenance (Right Care of the Infusion and Device), and timely removal (Right Therapy Duration and Device Removal). The aim is to demonstrate how individual technical interventions can be integrated into a coherent, system-based model of neonatal vascular access management, supporting the transition from reactive complication management toward proactive, patient-centered care across the entire catheter lifecycle.

## 2 Methodology

This narrative review summarizes the literature on the use, complications, and interventions associated with n-PICCs. A literature search was conducted using PubMed, Web of Science and Embase for articles published between January 2000 and February 2026. Search terms included: “neonate,” “infant,” “newborn,” “peripherally inserted central catheter,” “PICC,” “complications,” “thrombosis,” “occlusion,” “infiltration,” “catheter-related bloodstream infection,” “central line-associated bloodstream infection,” “CLABSI,” and “extravasation.” Relevant studies were identified and selected based on their relevance to the topic, and the findings were reviewed and discussed in a narrative format.

## 3 PICC: clinical practice

### 3.1 PICC introduction

#### 3.1.1 Key points for PICC insertion

PICC insertion should be performed by a specialized team, including trained physicians and strictly certified nurses, utilizing sterile technique within a dedicated aseptic workspace (5). The catheter length is measured from the puncture site to the midpoint of the sternal manubrium (22). Prior to PICC insertion, a portable ultrasound probe system is used clinically to screen for the optimal puncture vein. Chest x-ray is then employed to verify the catheter tip is positioned in the distal third of the superior vena cava (23). However, it is important to note that the infant's body position during x-ray can affect how the tip appears (24). Limb movement, such as shoulder adduction or elbow flexion, may shift the tip by 2–3 cm toward the heart, as demonstrated in prospective observational studies (25, 26). Therefore, a single static x-ray may not reflect the true tip location when the infant moves, highlighting the value of real-time positioning methods such as ultrasound or intracavitary electrocardiography.

**Figure 2:**
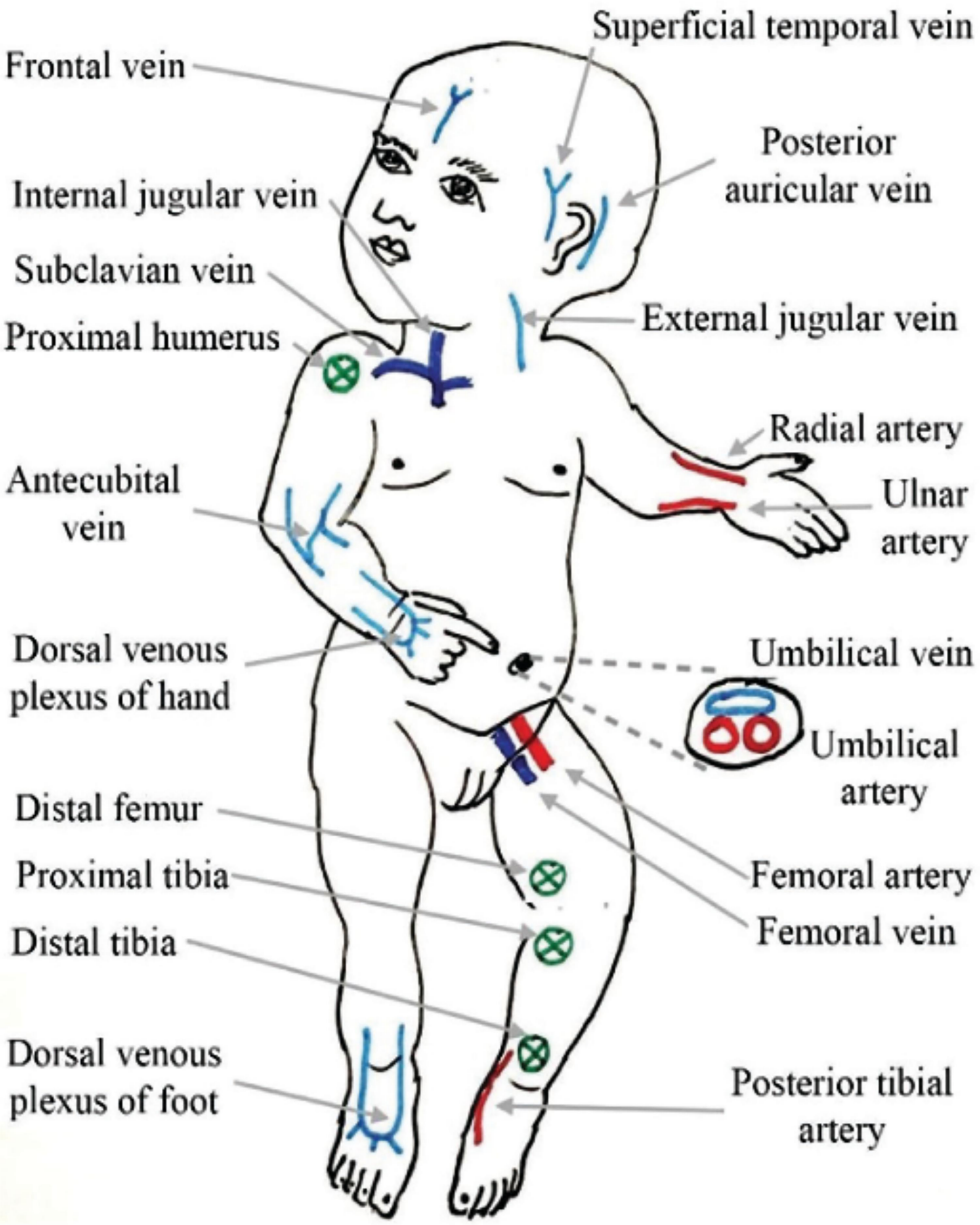
Common vascular access sites in neonates [adapted from vibhavari et al. (29)].

In neonates, a PICC can be inserted via any peripheral vein, with the catheter tip ultimately placed in the superior or inferior vena cava. Suitable insertion sites include the basilic vein and axillary vessels in the upper limbs; the great saphenous vein and popliteal vein in the lower limbs; and the postauricular and superficial temporal veins in the scalp (27). The most appropriate sites for PICC insertion are shown in Figure 2. A meta-analysis by Chen et al. (28) encompassing 17 studies and 4,790 neonates found that the overall complication rate for lower limb PICC was significantly lower than for upper limb PICC (OR = 0.83, 95% CI: 0.75–0.92). The right great saphenous vein was associated with a lower complication rate than the left. However, it is important to note that the 17 included studies were predominantly observational and single-center in design, with moderate heterogeneity (*I*^2^ = 45%). Therefore, while the findings suggest that the right great saphenous vein may be the preferred insertion site when conditions permit, the evidence should be interpreted with caution. Future prospective randomized trials are needed to confirm these findings and assess generalizability across different clinical settings.

#### 3.1.2 Catheter selection and maintenance

##### 3.1.2.1 Catheter materials and sizes

PICCs are primarily made of silicone or polyurethane materials. A review by Bahoush et al. (6) notes that polyurethane catheters, with advantages such as thin walls, large lumens, high strength, and good biocompatibility, have become the mainstream choice. The commonly used catheter diameters for n-PICCs range from 1 Fr to 2 Fr. In a single-center retrospective study by Avsar et al. (7), 92.3% of neonates received 1 Fr catheters. It is generally accepted that smaller catheters carry a lower risk of thrombosis but may increase the risk of occlusion, while larger catheters facilitate blood sampling and rapid fluid administration but are associated with a higher risk of thrombosis. However, these conclusions are primarily derived from retrospective studies and clinical experience. Definitions and monitoring methods for thrombosis and occlusion vary across studies, and no randomized controlled trials have directly compared the safety and efficacy of different catheter sizes. Therefore, catheter size selection should be individualized based on patient factors and treatment needs; current evidence does not support a universal recommendation for any specific size.

##### 3.1.2.2 Antimicrobial-impregnated catheters

Antimicrobial-impregnated catheters (miconazole and rifampicin-coated polyurethane catheters) have been developed to reduce central line-associated bloodstream infection (CLABSI) risk (14). However, studies have shown conflicting results. A retrospective study by Bayoumi et al. (15) comparing 1,242 standard PICCs with 791 antimicrobial catheters found no significant difference in CLABSI rates (1.11 vs. 1.86 per 1,000 catheter-days, *P* = 0.11). However, antimicrobial catheters had lower rates of accidental dislodgement, extravasation, occlusion, and phlebitis. In contrast, Avsar et al. (7) reported that antimicrobial catheters significantly reduced overall complication risks. Given that both studies are retrospective and no neonatal-specific RCT exists, these conflicting results preclude a strong recommendation. Their use should be considered on a case-by-case basis for high-risk infants, with awareness of higher costs and uncertain benefit.

##### 3.1.2.3 Catheter securement

Regarding catheter securement, current evidence suggests that sutureless securement techniques combined with chlorhexidine-impregnated transparent dressings or tissue adhesive can reduce the risk of catheter migration and accidental dislodgement compared with traditional adhesive tape. A scoping review by de Souza et al. (16) including 12 studies reported that after using tissue adhesive, PICC dislodgement decreased from 11.3% to 0.7%, and UVC dislodgement decreased from 24.6% to 7.7%. A randomized controlled trial by D'Andrea et al. (30) further confirmed that cyanoacrylate glue effectively reduces UVC dislodgement. However, most studies in the scoping review used tissue adhesive as part of a bundle intervention, making it difficult to assess its independent effect. Proper application is also important to avoid skin irritation. Overall, current evidence suggests that tissue adhesive may reduce dislodgement, but most data come from bundled interventions or observational studies, with only one RCT in umbilical catheters. Therefore, its independent efficacy and long-term safety in n-PICCs remain to be confirmed by high-quality RCTs.

##### 3.1.2.4 Flushing and locking

Regarding flushing and locking management, routine practice recommends flushing the PICC with 10 mL of normal saline immediately after each use and weekly when not in use (22). However, the choice between heparinized saline and normal saline remains debated. A Cochrane systematic review by Bradford et al. (31), which included multiple randomized controlled trials, found no significant difference between heparin and normal saline in preventing occlusion of central venous catheters in neonates, and heparin carries potential risks such as thrombocytopenia and drug incompatibility. A study protocol by Nascimento et al. (32) also noted a lack of high-quality evidence supporting routine heparin use. Therefore, current evidence suggests that normal saline may be the preferred flushing solution in the absence of clear indications. Thus, this conclusion requires further validation through large-scale, multicenter randomized controlled trials.

##### 3.1.2.5 N-PICCs insertion techniques

Currently, the puncture and catheter insertion are predominantly performed using the modified Seldinger technique, where the catheter is placed over a guidewire (33). A study comparing the conventional puncture technique with the modified Seldinger technique found that the latter significantly increased the puncture success rate and reduced associated complications (33). The main characteristics of common n-PICCs insertion techniques are summarized in the Table 1.

**Table 1:** Comparison of n-PICCs insertion techniques.

Technique	Key features	Advantages	Disadvantages
Modified Seldinger technique (MST)	Guidewire-guided insertion after venipuncture; often with ultrasound guidance	Higher first-attempt success rate; reduced vessel trauma; lower complication rate	Requires specialized training and equipment; longer procedure time initially
Traditional puncture technique	Blind venipuncture; direct catheter insertion without guidewire	Simple procedure; no special equipment needed	Lower first-attempt success rate; higher risk of multiple punctures; increased vessel damage
Guidewire-assisted technique	Guidewire inserted after successful venipuncture (same puncture site) to facilitate catheter advancement; no ultrasound guidance required	Easier catheter advancement; reduced resistance compared to direct insertion; no additional puncture	Limited evidence in neonates; requires pre-flush lubrication; may not improve first-attempt success rate (depends on initial venipuncture)

### 3.2 The timing for PICC placement

The optimal timing for PICC placement in neonates remains a subject of clinical debate. Traditionally, umbilical venous catheters (UVCs) are preferred for immediate vascular access in the first few days of life due to their rapid insertion at birth (1). However, prolonged UVC dwell time (>7–10 days) is associated with an increased risk of infection, thrombosis, and hepatic complications (34, 35). Therefore, when central access is expected to be needed beyond the first week, early replacement with a PICC may be considered. Several studies have explored the optimal timing for transitioning from UVC to PICC. Hess et al. (36) surveyed 12 NICUs and found that UVC dwell times ranged from 1 to 10 days, while PICC dwell times ranged from 1 to 14 days. The reported infection rates were 4.2% for UVC and 15.0% for PICC, suggesting that the decision to replace UVC with PICC should balance the risks of prolonged UVC use against the risks of PICC insertion. Primary PICC placement (without prior UVC) is often indicated when long-term central access is anticipated from the outset, such as in extremely low birth weight infants requiring prolonged parenteral nutrition, or in infants with surgical conditions where UVC placement is contraindicated or may interfere with planned procedures (7, 22). In these cases, PICC serves as the primary central venous access device rather than a replacement. The decision regarding the timing and need for PICC as replacement should be individualized based on patient characteristics, anticipated duration of therapy, and institutional protocols. Table 2 summarizes the key considerations.

**Table 2:** Timing and indications for PICC as replacement vs. Primary Placement.

Aspect	PICC as replacement (after UVC)	Primary PICC placement
Typical timing	3–10 days after birth	First few days of life
Indications	Prolonged need for central access beyond UVC dwell time; UVC complications (malposition, infection, thrombosis)	Long-term therapy anticipated from birth; UVC contraindicated (e.g., omphalocele, necrotizing enterocolitis); need to preserve umbilical site for future procedures
Considerations	Balance risk of extended UVC use vs. risk of new PICC insertion; potential for increased painful procedures	Avoids UVC-related complications; may require more skilled insertion in smaller infants
Evidence	Ongoing RCT evaluating non-inferiority of extended UVC dwell time	Observational studies support feasibility in ELBW infants

### 3.3 Indications for n-PICCs

The primary indications for n-PICCs include preterm or very low birth weight infants requiring prolonged parenteral nutrition support. They also include critically ill infants needing continuous infusion of hyperosmolar or irritating medications, and infants with poor peripheral venous access precluding the establishment of reliable peripheral intravenous lines (1, 6, 22).

### 3.4 Contraindications, high-risk factors, and related precautions for PICC insertion

For n-PICCs placement, the following relative contraindications and high-risk factors must be fully considered. In neonates who have undergone abdominal surgery (e.g., gastroschisis, necrotizing enterocolitis), lower limb venous catheterization can significantly increase the risk of severe thrombotic complications due to impaired venous return from postoperative intra-abdominal hypertension. Therefore, it should be chosen cautiously with close monitoring of the catheter tip position (37). Selection of the appropriate insertion site is critical. The femoral vein lies close to the perineum, which raises the infection risk, so should be avoided in this site when possible (38). If lower limb placement is necessary, the right great saphenous vein, with its straighter anatomical course, is a preferable choice, as it is associated with a lower incidence of catheter occlusion compared to the left side (28). The patient's own condition is equally important: severe coagulopathy increases the risk of thrombosis or bleeding (39). The procedure should be postponed until vital signs are stabilized in extremely unstable conditions (e.g., severe shock, hypothermia) (22). Furthermore, a larger catheter size is not necessarily better; a lumen diameter >3 Fr increases thrombosis risk, and the smallest possible size that meets therapeutic requirements should be selected (6). Even with correct initial placement, the catheter may shift due to the infant's limb movements or growth. For this reason, regular imaging checks are recommended to monitor the tip position and help prevent serious complications like pericardial tamponade.

### 3.5 PICC insertion procedures

Standardized procedures for PICC insertion are essential to safeguard and optimize high-quality nursing practice, as well as to improve patient safety and well-being. Table 3 serves as a fundamental basis for developing targeted nursing protocols, management policies, and operational standards.

**Table 3:** Standardized procedures, policies, and steps to promote safety and comfort in neonatal patients .

Step	Content
1	Prior to the procedure, the primary purpose of the operation shall be clearly explained to the parents or guardians of the pediatric patient, so that they understand the overall steps and rationale, thereby facilitating their comprehension of the entire procedure (6, 40).
2	In accordance with institutional policies, all mandatory procedures and equipment management must be performed by highly qualified professionals, such as interventional radiologists, anesthesiologists, and operating room specialist nurses (20, 41).
3	For effective skin preparation, chlorhexidine should be used prior to catheter insertion. Meanwhile, to prevent and control infection, the skin around the puncture site should be covered with a sterile drape (6). In this regard, gloving, gowning, masking, and hand hygiene represent core elements of care provision (11, 17, 42).
4	First, a tourniquet is placed under the infant's shoulder, an appropriate sterile gel is applied to the puncture site, and ultrasound guidance is then used to optimize the venous access pathway for the target vein (10, 43).
5	Under strict aseptic conditions, 1% lidocaine is administered intradermally; under ultrasound guidance, the puncture site is enlarged with a scalpel, and a fine needle is inserted followed by advancement of a guidewire through the needle. Once blood return is observed, the tourniquet should be released immediately and the needle withdrawn, and the PICC is finally inserted over the guidewire into the superior vena cava (SVC) (33, 44, 45).
6	The guidewire is removed once securely positioned in the patient, and an injection cap is then connected to the catheter hub (6).
7	The catheter is measured and flushed with normal saline (NS) to confirm blood return; meanwhile, chest x-ray is performed to verify that the catheter tip is located in the distal third of the superior vena cava (SVC) (6, 46).
8	Finally, the catheter insertion site is secured using a sutureless stabilization technique and covered with a chlorhexidine-impregnated transparent dressing (16, 30, 47).
9	Overall postoperative management of pediatric patients is essential for maintaining PICC patency and preventing various complications; strict aseptic hand hygiene shall be implemented throughout the entire process, including dressing changes, handling of contaminated dressings, medication administration, and intravenous infusion (17, 18).
10	The patient's vital signs and symptoms should be documented for appropriate monitoring of the clinical condition, evaluation of the overall status, and communication with other nursing staff, so as to provide adequate care and prevent PICC-related complications (20, 48, 49).

Adapted from Sona et al. (50) and Bahoush et al. (6).

## 4 Major complications

### 4.1 Overview of PICC-related complications

PICC is widely used for long-term IV access in newborns, but it carries risks. The overall complication rate ranges from 10% to 40% (7, 36). Complications may lead to unplanned catheter removal, prolonged hospital stay, increased costs, and even death (51, 52). Infections are the most common, with CLABSI rates of 1.46–15.0 per 1,000 catheter-days, especially in very low birth weight infants (8, 38). Thrombosis occurs in 0.8%–4.3% of cases, but can reach 16% in infants undergoing abdominal surgery (7, 37, 39). Mechanical complications, such as phlebitis and occlusion, occur in 10.7%–36.7% of cases (7, 36). Cardiac tamponade is rare (0.07%–2%) but life-threatening (9, 53). The key characteristics and incidence ranges of major complications are summarized in Table 4, providing a foundation for subsequent analysis of risk factors and preventive strategies.

**Table 4:** Incidence and characteristics of Major n-PICCs complications.

Complication type	Incidence range	Key characteristics	Key contributing factors
Infective complication (8, 17, 38)	1.46–15.0 per 1,000 catheter-days	Most common serious complication; can lead to sepsis	Low birth weight, small gestational age, prolonged dwell time, femoral vein placement
Thrombosis formation (7, 37, 39)	0.8%–4.3% (clinically overt)	May cause catheter dysfunction, limb swelling	Non-central tip position, larger catheter gauge, abdominal surgery, infection
Mechanical failure (7, 36, 54)	10.7%–36.7%	Includes phlebitis, occlusion, dislodgement, breakage	Mechanical irritation, catheter size, securement method, insertion site
Pericardial Tamponade and Pleural Effusion (9, 25, 53)	0.07%–2%	Rare but highly fatal; requires emergency management	Tip in right atrium, catheter migration

### 4.2 Infective complication

Infection is one of the most common and serious complications associated with PICC. Multiple studies have reported the incidence of PICC-related infections in neonates, but results vary considerably across studies. A retrospective analysis by Xu et al. (8) of 446 neonates reported CLABSI and catheter-related bloodstream infection (CRBSI) rates of 5.6 and 1.46 per 1,000 catheter-days, respectively. Hu et al. (38) reported a CRBSI rate of 10.62% in 386 neonates, while Zhang et al. (39) reported 4.74% in 680 neonates. Wang et al. (17) reported a CRBSI rate of 3.1 per 1,000 catheter-days in very low birth weight infants. A survey by Hess et al. (36) across 20 NICUs in Austria and Germany reported a PICC-associated infection rate as high as 15.0 ± 3.4% in very low birth weight infants. Notably, most of these studies were single-center retrospective designs, and definitions of infection, surveillance methods, and study populations varied across studies, resulting in a wide reported incidence range (1.46–15.0 per 1,000 catheter-days). Among them, the multicenter survey by Hess et al. (36) reflects practice variations across different centers and reported a higher infection rate, suggesting heterogeneity in infection prevention practices. In contrast, the lower infection rate reported by Wang et al. (17) following bundle intervention indirectly supports the effectiveness of standardized care measures. Therefore, directly comparing infection rates across studies is of limited value; greater attention should be paid to the practice factors contributing to these differences.

Throughout this review, two related but distinct infection metrics are used. Central line-associated bloodstream infection (CLABSI) is a surveillance definition used by the CDC/NHSN, referring to a primary bloodstream infection in a patient with a central line within 48 h before infection onset, without requiring positive catheter tip culture. Catheter-related bloodstream infection (CRBSI) is a clinical definition requiring both positive peripheral blood culture and positive semi-quantitative catheter tip culture (>15 CFU) from the same organism, with no other identifiable infection source. In neonates, the distinction is clinically relevant because CRBSI provides stronger evidence of catheter causality but requires catheter removal for diagnosis, whereas CLABSI is more practical for surveillance but may overestimate catheter-attributable infections. In this review, both terms are reported as used in the original studies; where studies used CLABSI or CRBSI, we retain the original terminology to accurately reflect the evidence base.

Regarding risk factors for infection, multivariate analyses have consistently identified low birth weight, prolonged PICC dwell time, and a 5-min Apgar score ≤7 as independent risk factors for CRBSI (8, 38, 39). Lower gestational age (38) and femoral vein catheterization (38) also significantly increase infection risk. Xu et al. (8) further found that head/neck catheterization was associated with a significantly lower infection rate compared to upper or lower limb placement, while prolonged antibiotic use significantly increased infection risk. However, evidence for these risk factors is primarily derived from retrospective analyses and requires validation in prospective studies. The high infection risk associated with femoral vein catheterization is attributed to its anatomical proximity to the perineum and susceptibility to contamination.

### 4.3 Thrombosis formation

Thrombosis is a significant complication associated with n-PICCs, and its reported incidence varies considerably due to differences in diagnostic criteria, study populations, and monitoring frequency. In a single-center retrospective study of 610 neonates, Avsar et al. (7) reported a clinically overt thrombosis rate of 0.8%. A multicenter survey by Hess et al. (36) reported a PICC-related thrombosis rate of 4.3 ± 3.5% in very low birth weight (<1,250 g) infants, with wide variation across centers (0%–10%). In a study focusing on neonates undergoing abdominal surgery, Kisa et al. (37) reported a severe thrombotic complication rate as high as 16% with lower limb PICCs. Differences in diagnostic methods (clinically overt vs. imaging-based screening) and monitoring frequency across studies contribute to the wide incidence range (0.8%–16%).

Thrombus formation is influenced by multiple factors. Catheter tip position is a key factor, as a non-central tip within a vessel with slow blood flow can lead to flow disturbance and endothelial injury. In the study by Kisa et al. (37), the catheter tip was positioned at or below L1 in all 5 neonates with severe thrombosis, highlighting the importance of ensuring a central tip position, though this conclusion is based on a small retrospective sample and requires prospective validation. Insertion site significantly impacts thrombotic risk: for neonates undergoing abdominal surgery, lower limb PICC carries a substantially increased risk due to impaired venous return from postoperative intra-abdominal hypertension (37); right lower limb placement is associated with a lower rate of catheter occlusion compared with the left, likely due to the straighter anatomical course of the right common iliac vein (28). Catheter size is also associated with thrombosis, with larger diameters (>3 Fr) increasing risk (6, 7); however, current evidence is primarily observational, and no randomized controlled trials have directly compared the safety of different catheter sizes. Additionally, infection and thrombosis have a synergistic effect. In the study by Kisa et al. (37), all 5 neonates with severe thrombosis had positive blood cultures, and 2 required prolonged antibiotic therapy for septic thrombophlebitis. A case report by Zhang et al. (39) described a neonate with fungal septicemia who developed concurrent cardiac and renal artery thrombosis, further supporting that coagulopathy during infection significantly increases thrombotic risk, though this conclusion is based on a case report and requires larger studies for confirmation.

### 4.4 Mechanical failure

Mechanical complications are a relatively common type of complication associated with n-PICCs, primarily including catheter occlusion, phlebitis, catheter damage, and catheter dislodgement. In a single-center retrospective study of 610 neonates, an overall incidence of mechanical complications of 36.7%, with phlebitis being the most frequent (14.1%), followed by catheter occlusion (7.0%), catheter damage or leakage (3.8%), and catheter dislodgement (2.3%) (7). Li et al. (22) reported phlebitis in 4.25% and catheter occlusion in 2.04% of cases. Yilmaz et al. (55) described a rare case of intravascular knot formation in a preterm infant's PICC, while He et al. (56) reported a case of difficult catheter removal due to intravascular catheter folding. These incidence data are derived primarily from single-center retrospective studies, and differences in case composition, diagnostic criteria, and monitoring frequency across studies contribute to a wide reported range (2.04%–36.7%). Rare complications are mostly reported as case reports; while they provide important clinical insights, they do not allow for accurate estimation of true incidence rates.

The occurrence of mechanical complications is influenced by multiple factors. Phlebitis is closely related to mechanical irritation. In a single-center observational study, Igarashi et al. (54) found that after discontinuing routine palpation of the puncture site, the incidence of phlebitis decreased from 34.5% to 3.2%, suggesting that minimizing unnecessary manipulation of the insertion site is an effective preventive measure. However, this conclusion is based on a single-center study and requires validation in larger populations. Catheter size selection is crucial; an excessively large diameter increases the risk of vascular injury, and the smallest possible size that meets therapeutic requirements should be chosen (6, 7). Insertion site also affects complication rates. A study by Chen et al. (28) found that right lower limb placement was associated with a lower rate of catheter occlusion compared with the left, and great saphenous vein cannulation had a higher first-attempt success rate than femoral vein cannulation (90.77% vs. 63.64%). However, this was a single-center study, and findings need to be validated in larger populations. Rare complications such as catheter folding and knot formation are often associated with vascular anatomical variations or improper technique (55, 56), highlighting the importance of familiarity with vascular anatomy and avoiding forceful catheter advancement.

### 4.5 Pericardial tamponade and pleural effusion

Pericardial tamponade and pleural effusion are among the most severe complications of n-PICCs. Although the incidence ranges from 0.07% to 2%, the associated mortality is exceedingly high, warranting significant clinical attention. A retrospective analysis by Zareef et al. (9) of 810 n-PICCs cases over 10 years reported 4 cases (0.49%) of severe pleural/pericardial effusion, all of whom survived following timely bedside ultrasound diagnosis and intervention. In a study by Trinh et al. (57) of 207 pediatric patients with PICC over one year, pericardial effusion was identified in 6 cases (2.9%), with 4 (1.9%) progressing to pericardial tamponade; all were successfully managed with prompt diagnosis and intervention. A literature review by Hou et al. (53) of 24 cases of PICC-related pericardial tamponade showed an overall mortality rate of 37.5%, which decreased to 6.25% in patients who underwent pericardiocentesis. It is worth noting that most of this evidence comes from single-center retrospective analyses or case series, and reported incidence rates vary considerably (0.49%–2.9%), likely due to differences in study populations, monitoring methods, and diagnostic criteria.

The occurrence of pericardial tamponade is influenced by multiple factors, with the position of the catheter tip being the most critical risk factor. An analysis by Hou et al. (53) revealed that in 79.2% of pericardial tamponade cases, the catheter tip was located within the right atrium, and in 8.3% of cases, it was positioned at the cavoatrial junction. Catheter migration is another significant contributing factor, occurring in approximately 29.2% of cases where the catheter moved from its initial central position into the atrium. A prospective observational study by Nadroo et al. (25) found that upper extremity PICC lines can migrate inward due to limb movement, with the highest risk occurring when shoulder adduction and elbow flexion happen simultaneously, bringing the catheter tip closest to the atrium. A study by Gupta et al. (58) also confirmed that catheter migration is a major cause of pericardial tamponade. However, evidence for these risk factors is primarily derived from observational studies with small sample sizes and requires validation in larger prospective studies. The underlying mechanisms primarily involve two aspects: first, direct mechanical injury from the catheter tip, where repeated friction against the atrial wall leads to myocardial perforation; and second, chemical injury caused by hyperosmolar nutrient solutions entering the pericardial cavity either through the damaged myocardium or via direct permeation.

## 5 Preventive measures for major PICC-related complications

### 5.1 Technical interventions

#### 5.1.1 Ultrasound-guided insertion

Ultrasound guidance has been widely adopted as a technique for improving n-PICCs insertion success, with real-time point-of-care ultrasound (POCUS) playing a particularly important role. Traditional landmark-based methods often fail, especially in very low birth weight infants. Real-time POCUS allows dynamic visualization of the vein—including location, course, and diameter-during the procedure, enabling operators to adjust the puncture angle and depth in real time, select the optimal puncture site, significantly increase first-attempt success, and reduce vascular injury from repeated punctures (59). Studies show that real-time ultrasound guidance can raise first-attempt success rates from 0% to 64% to over 90%, shorten procedure time from 33 min to 5 min, and reduce complications such as malposition and phlebitis (11, 59). In clinical practice, real-time POCUS is used not only for pre-insertion vessel assessment and puncture guidance, but also for real-time navigation of the catheter tip during insertion. By dynamically monitoring the tip position, operators can promptly detect and correct malposition, avoiding tip entry into the right atrium or branch vessels (10). After insertion, ultrasound can also be used for immediate tip confirmation. Compared with traditional x-ray, ultrasound offers advantages such as no radiation exposure, repeatability, and real-time dynamic visualization, serving as an effective complement or alternative to x-ray (60). Standardized protocols and systematic training are essential for effective use. The Neo-ECHOTIP protocol proposed by Barone et al. (10) provides a structured approach to real-time ultrasound-guided central venous access in neonates. A blended training program combining theory and simulation significantly improves nurses’ real-time ultrasound skills and insertion success rates. Despite the compelling advantages of real-time POCUS, the evidence base requires careful interpretation. The reported success rates are highly operator-dependent, and structured training is essential for replicating outcomes from specialized centers (61). Furthermore, few studies have evaluated the long-term vascular consequences of ultrasound-guided multiple punctures in neonates (62). Therefore, while ultrasound guidance is a reasonable adjunct to improve insertion accuracy, its effectiveness in reducing overall complications in routine practice depends on operator competency and protocol adherence; further multicenter trials are needed to confirm its superiority over landmark techniques for hard outcomes.

#### 5.1.2 Intracavitary electrocardiography for tip positioning

Intracavitary electrocardiography (IC-ECG) uses the saline column within the catheter as an exploring electrode to determine tip position by monitoring real-time changes in *P*-wave amplitude. The P-wave amplitude increases when the tip reaches the superior vena cava, peaks at the cavoatrial junction, and decreases or becomes negative upon entering the right atrium (63). This technique enables real-time tip positioning during insertion, avoiding repeated x-ray adjustments, and significantly reduces complications such as malposition, phlebitis, and arrhythmias (12, 13). Clinical studies have shown that IC-ECG has an applicability of 97%, a first-attempt success rate of 91%, and an overall success rate of 98%, reducing the risk of overall complications by 77% (12). It is particularly suitable for very low birth weight infants, reducing radiation exposure and procedure time. Application requires stable ECG waveforms to avoid interference from infant agitation or respiration. It may be considered as a routine adjunct in centers with appropriate equipment and expertise. Although IC-ECG demonstrates high feasibility and accuracy, its applicability may be reduced in neonates with arrhythmias, poor skin-electrode contact, or unstable ECG signals due to respiratory distress or agitation (64). Moreover, the technique requires a dedicated device, which may not be readily available in all NICUs, and most published evidence comes from single centers with experienced operators (65). Thus, while IC-ECG appears to be a valuable adjunct in centers with experience, its routine adoption should be accompanied by local validation; the evidence is still preliminary and operator-dependent.

#### 5.1.3 Tissue adhesive for catheter securement

Tissue adhesive (cyanoacrylate) has emerged as a novel material for securing vascular access devices in neonates, offering an alternative to traditional sutures or tape. A scoping review by de Souza et al. (16) reported significant reductions in dislodgement rates (PICC: from 11.3% to 0.7%; UVC: from 24.6% to 7.7%), along with decreased bloodstream infection (UVC: from 7.7% to 3.1%) and phlebitis (from 13% to 3%) rates. An observational study by van Rens et al. (66) further confirmed reduced dislodgement without increased infection. However, the evidence requires critical appraisal: most studies were observational or quasi-experimental, and tissue adhesive was typically implemented as part of a multifaceted bundle, making it difficult to isolate its independent effect (16). Only one RCT has evaluated cyanoacrylate for UVC securement, and no similar trial exists for PICC securement (30). The retrospective, single-center design of van Rens et al. (66) without a concurrent control group limits causal inference, and the reported reductions in infection and phlebitis may reflect overall bundle effects rather than the specific contribution of tissue adhesive. Moreover, long-term skin safety in premature infants with immature skin barriers remains inadequately studied. Proper application technique is essential to avoid adhesive entering the puncture site or causing skin irritation. Therefore, while tissue adhesive is a promising securement method—particularly for neonates with high mobility or increased dislodgement risk—high-quality RCTs with adequate sample sizes and long-term safety outcomes are needed before it can be recommended as a universal standard of care.

#### 5.1.4 Bundle care strategies

Bundle care strategies combine multiple evidence-based interventions to achieve better outcomes than single measures. The central venous catheter bundle proposed by the Institute for Healthcare Improvement consists of five core elements: hand hygiene, maximal sterile barrier precautions, chlorhexidine skin disinfection, optimal puncture site selection, and daily assessment of catheter necessity (67). For n-PICCs practice, Wang et al. (17) extended this framework by incorporating a PICC treatment room, dedicated dressing management, and standardized flushing and locking protocols tailored for very low birth weight infants, reporting a reduction in catheter-related infection from 10.0 to 2.2 per 1,000 catheter-days. Similarly, Zhu et al. (18) applied an evidence-based continuous quality improvement approach and reported a reduction in overall PICC-related complications from 46.10% to 15.45% over four years. However, both studies should be interpreted with caution: Wang et al. (17) employed a single-center case-control design, and the observed reduction may be confounded by temporal trends, concurrent quality improvement initiatives, and changes in case mix; Zhu et al. (18) was a single-center retrospective analysis without a concurrent control group, and the reported improvement may reflect secular trends rather than the causal effect of the intervention alone. Moreover, the individual contribution of each bundle element remains unknown, as most studies implemented the bundle as a single package, making it difficult to determine which elements are essential and which may be modified or omitted without compromising outcomes. Bundle compliance was also often measured as a binary (all-or-none) indicator, which may obscure the relationship between partial adherence and outcomes. Future research should employ stepped-wedge or cluster randomized designs to strengthen causal inference and should evaluate the minimum effective bundle elements to reduce unnecessary care burden. Based on current evidence from before–after studies, it is reasonable to apply bundle strategies throughout insertion and maintenance, supported by monitoring-feedback-improvement to ensure implementation quality; however, the independent contribution of each element remains unclear.

#### 5.1.5 Emerging concepts in continuous monitoring of catheter function

Traditional monitoring of n-PICCs has focused primarily on tip position confirmation via x-ray or ultrasound, and clinical observation of the insertion site and limb swelling. However, recent developments in infusion dynamics and low-flow physiology have highlighted the importance of continuous functional monitoring of catheter patency and infusion reliability (68). Including occlusion, thrombosis, and infiltration, catheter dysfunction often develops progressively rather than occurring abruptly. Early detection of these subclinical changes is critical for preventing complete catheter failure and associated complications. Emerging technologies enable real-time monitoring of infusion pressures and flow dynamics, allowing clinicians to detect increases in resistance or changes in flow patterns that may indicate early catheter dysfunction. In neonatal populations, where catheters are small-caliber (1–2 Fr) and infusion rates are often low, even subtle changes in infusion dynamics can signal impending occlusion or thrombosis. Preliminary studies suggest that continuous flow monitoring systems may detect catheter dysfunction earlier earlier than traditional clinical assessment, potentially enabling preemptive intervention before complete occlusion or extravasation occurs (69). These systems are particularly valuable in the NICU setting, where neonates may have limited physiological reserve and where catheter failure can rapidly lead to treatment interruption. Future directions in monitoring include the integration of artificial intelligence-assisted algorithms that can analyze infusion pressure waveforms and predict catheter dysfunction before clinical symptoms manifest. Such intelligent monitoring systems could transform neonatal vascular access management from a reactive paradigm to a proactive paradigm. However, evidence for these emerging technologies in neonates remains limited, and further research is needed to establish their clinical utility, safety, and cost-effectiveness.

### 5.2 Organizational interventions

#### 5.2.1 Specialized team building

The establishment of specialized PICC teams is widely regarded as an important strategy for ensuring the safety of neonatal vascular access and reducing complication rates. Van Rens et al. (19) introduced the concept of the “n-PICCs subspecialty,” emphasizing that PICC insertion is not merely a technical procedure but an art requiring specialized training, which should be performed by systematically trained professionals. This team typically comprises neonatologists, neonatal nurse practitioners, and NICU specialist nurses. Through clear division of roles and collaboration, they establish standardized procedures for catheter insertion and maintenance (41, 56). Multidisciplinary collaboration holds unique value in PICC management. Bayoumi et al. (41) evaluated the effect of implementing an Epicutaneo-Caval Catheter team, finding that the introduction of this team-based model significantly improved catheter insertion success rates and reduced complication rates. This team worked closely with the specialized neonatal nursing unit, jointly responsible for vascular access device selection, assessment of insertion indications, catheter maintenance, and data registry. Although specialized vascular access teams are widely regarded as a cornerstone of safe PICC practice, the evidence base requires careful interpretation. A systematic review concluded that while teams are a promising intervention, the level of evidence for effectiveness is low, with all included studies being observational and reporting a relative CLABSI decrease of 45%–79% (70). Similarly, the study by Bayoumi et al. (20), which demonstrated improved insertion success and reduced complications, employed a pre-post design without a concurrent control group, and the observed benefits may be attributable to secular trends or concurrent interventions rather than team structure alone. Moreover, the optimal composition, staffing ratios, and training requirements for neonatal vascular access teams have not been established through rigorous comparative studies, and cost-effectiveness analyses of team-based models remain scarce. Although specialized teams are widely recommended, the evidence for their effectiveness is based on observational studies with high risk of confounding. Future research should compare different organizational models and evaluate their feasibility, sustainability, and cost-effectiveness.

#### 5.2.2 Nursing training

Nursing training encompasses four key aspects: standardized training, simulation training, competency certification, and continuous quality improvement. Standardized training is key to enhancing the quality of PICC care. The ultrasound-guided PICC insertion nurse training program designed employed a blended learning model that integrated online theoretical instruction with in-person simulation training (59). This approach significantly improved nurses’ ultrasound skills and catheter insertion success rates. The training curriculum covered fundamental ultrasound principles, vascular anatomy, puncture techniques, and complication recognition. Competency for independent practice was ensured through theoretical, procedural, and clinical practice assessments. Simulation training is an effective method for enhancing PICC procedural skills. Bayoumi et al. (71) reported on a five-year neonatal simulation training program in Qatar, which utilized a standardized curriculum and simulated scenario training to significantly improve the catheter insertion success rate and confidence among team members. This training followed the “Learn, See, Practice, Prove, Do, Maintain” six-step pedagogical framework, encompassing theoretical knowledge acquisition, hands-on simulation practice, and guided clinical practice, ensuring trainees progressively master the operational skills. Competency certification is a critical component in ensuring the quality of PICC care. A study indicated that hospitals with specialized PICC teams place greater emphasis on nurse credentialing and ongoing training (72). Competency certification should encompass theoretical assessment, simulation-based skill evaluation, and clinical practice assessment, and requires periodic re-evaluation to ensure the continuous updating of nursing skills. Van Rens et al. (19) emphasized that new personnel should follow a rigorous competency certification pathway, completing theoretical learning, simulation training, clinical observation, and supervised practical operation before performing independent catheter insertion. However, the evidence for these training interventions has methodological limitations. Both the blended learning program reported by Escobar Gimenes et al. (59) and the simulation program by Bayoumi et al. (71) employed pre-post designs without control groups, leaving observed improvements confounded by concurrent factors such as cumulative experience and changes in case mix. Furthermore, optimal training content, duration, and refresher frequency have not been established through comparative studies, and the impact of specific training models on patient-centered outcomes—including pain and family experience—remains poorly defined. Future research should employ standardized outcome measures to validate competency assessment tools against clinically meaningful endpoints, as current evidence on training effectiveness is mostly preliminary and uncontrolled.

### 5.3 System-level interventions: continuous quality improvement

#### 5.3.1 Evidence-based quality improvement models

Evidence-based quality improvement combines evidence-based practice with continuous quality improvement, using multidisciplinary collaboration, phased implementation, and ongoing monitoring to achieve sustained improvements in clinical outcomes. Zhu et al. (18) applied this model to PICC management over a four-year period. Through establishing a multidisciplinary team, synthesizing evidence, implementing in phases, and providing regular feedback, they reduced overall PICC-related complications from 46.10% to 15.45% and unplanned removals from 24.03% to 11.82%. The study demonstrated that meaningful improvement requires 18–24 months of sustained effort, highlighting the importance of long-term commitment. Key lessons from high-impact quality improvement studies can be summarized as follows: first, establishing a monitoring-feedback-improvement loop is essential, using regular data analysis to identify problems and guide targeted interventions; second, multidisciplinary collaboration is critical for driving process optimization and requires breaking down professional silos to build consensus; third, standardized procedures and compliance monitoring must go hand in hand—developing protocols alone is insufficient, and checklists are needed to ensure adherence; fourth, ongoing feedback and retraining are crucial for sustaining improvement. These insights provide a practical foundation for developing a comprehensive vascular access safety management system.

## 6 Clinical implications and future directions

### 6.1 Key clinical implications

The prevention of PICC-related complications in neonates can be summarized at four levels. At the technical level, ultrasound guidance and intracavitary electrocardiography may be considered as routine techniques to improve insertion accuracy and reduce tip-related complications. At the material level, tissue adhesive may be considered for use to effectively reduce catheter dislodgement; antimicrobial-impregnated catheters may be considered for high-risk infants but are not recommended for routine use. At the management level, bundle care strategies should be implemented throughout the insertion and maintenance process, supported by standardized checklists to ensure adherence; establishing specialized vascular access teams may be beneficial. At the system level, continuous quality improvement mechanisms are central to achieving sustained reductions in complications, requiring the establishment of a monitoring-feedback-improvement loop. These findings suggest that neonatal vascular access management should shift from a single-intervention approach toward a system-based approach. Integrating technical optimization, material selection, standardized procedures, and organizational management into a comprehensive safety management system covering the full process-assessment, insertion, maintenance, removal, and monitoring-represents a key pathway for reducing complications and ensuring patient safety. Future research should further validate the feasibility and scalability of such a system across different levels of healthcare settings.

### 6.2 Clinical management pathway

Based on the evidence presented above, this section proposes a clinical management pathway for n-PICCs, encompassing six key stages: pre-insertion assessment, insertion procedure, tip confirmation, daily maintenance, removal decision, and post-removal observation, as shown in Table 5.

**Table 5:** Clinical management pathway for n-PICCs.

Stage	Objectives	Core interventions	Monitoring	Expected outcomes
1. Pre-insertion	Identify high-risk neonates; select appropriate device and site	Risk stratification (BW, GA, surgery history); Doppler ultrasound for vein mapping (8, 37, 38)	Coagulation profile; vessel diameter and patency	Reduced insertion failure; appropriate device selection
2. Insertion	Maximize first-attempt success; minimize vessel trauma	Real-time POCUS; IC-ECG tip navigation; Modified Seldinger technique (10, 11, 33)	Real-time tip P-wave changes; needle visualization	>90% first-attempt success; reduced malposition (12)
3. Securement & Maintenance	Prevent dislodgement and infection	Tissue adhesive securement; CHG-impregnated dressing; Standardized flushing protocol (NS) (16, 17, 31)	Daily site inspection; external catheter length measurement	Reduced dislodgement (from 11.3% to 0.7%) (16); lower CLABSI
4. Monitoring & Removal	Early detection of dysfunction; timely removal	Continuous infusion dynamics monitoring; Daily necessity assessment (54, 63)	Pressure waveform changes; limb swelling; fever	Prevention of cardiac tamponade; reduced unplanned removals

### 6.3 Research gaps

Despite significant progress, several evidence gaps persist. First, high-quality randomized controlled trials are urgently needed to validate the independent efficacy of antimicrobial-impregnated catheters and tissue adhesive in neonates, as most current data come from bundled interventions or retrospective analyses. Second, robust epidemiological data on rare but catastrophic complications, such as cardiac tamponade and catheter-related thrombosis, are lacking, and no validated risk prediction models exist for the neonatal population. Third, the long-term vascular consequences of n-PICCs placement remain largely unexplored, as most studies focus on short-term outcomes. Fourth, standardized, validated competency assessment tools for PICC operators are not yet established across institutions. Fifth, comprehensive cost-effectiveness analyses comparing different prevention strategies are scarce, limiting informed resource allocation in resource-limited settings. Addressing these gaps will require collaborative, multicenter efforts and standardized data collection.

## 7 Discussion

The findings of this review demonstrate that n-PICCs-related complications remain a significant clinical challenge, with incidence rates ranging from 10% to 40% across studies (7). While numerous evidence-based interventions, including ultrasound guidance, intracavitary electrocardiography, tissue adhesive securement, and bundle care strategies have demonstrated clinical value in reducing specific complications, the persistent variation in outcomes across centers and the continued occurrence of complications despite technological advances raise important questions about the gap between evidence and practice.

### 7.1 Why complications persist despite technological advances

The persistence of n-PICCs-related complications despite the availability of effective preventive strategies can be understood through multiple lenses. However, it is important to note that the evidence base for many of these strategies remains moderate to low, and their effectiveness in real-world practice is often less robust than reported in single-center studies. First, most evidence for individual interventions comes from single-center observational studies with limited generalizability (70, 73). Second, the implementation of evidence-based interventions is often fragmented rather than integrated into a coherent system of care (21). Third, and perhaps most importantly, the translation of evidence into routine clinical practice is hindered by human factors and system-level barriers that are rarely addressed in clinical research (74).

### 7.2 The shift from reactive to proactive vascular access management

A fundamental paradigm shift is emerging in neonatal vascular access: from reactive complication management toward proactive, preventive care across the entire catheter lifecycle. This shift requires moving beyond the question of “which single intervention works” to “how do we build a system that consistently delivers safe vascular access care.” The 7-Rights Framework provides a conceptual scaffold for this transition, organizing the full spectrum of vascular access activities into a coherent, patient-centered model (21). Rather than viewing ultrasound, IC-ECG, tissue adhesive, and bundles as independent interventions, this framework positions them as interconnected components of a comprehensive safety system, each addressing a specific domain of vascular access care.

### 7.3 Human factors and clinical inertia

The review has focused predominantly on technical interventions, yet human factors increasingly determine outcomes in vascular access care. Clinical inertia represents a significant barrier to the adoption of evidence-based practices. In the context of neonatal vascular access, van Rens et al. have recently articulated how “success becomes a barrier” (74). Once a practice becomes associated with improvement, it acquires near-sacred status, establishing a compliance mindset that makes change harder. Innovations struggle to enter routine practice even when supported by evidence because deviation from established practice may be perceived as introducing risk rather than addressing emerging harms. This phenomenon, termed “validation freeze” and “safety conservatism,” helps explain why evidence-based interventions such as tissue adhesive securement and real-time infusion monitoring have not been universally adopted despite promising data.

### 7.4 Implementation science and barriers to change

The gap between evidence and practice in neonatal vascular access is not simply a knowledge deficit; it reflects broader implementation challenges (73). Key barriers include (1) organizational culture: legacy protocols, regulatory conservatism, and professional comfort with familiar practices inhibit meaningful evolution; (2) workforce factors: variation in training, competency, and turnover affects consistency of care; (3) resource constraints: many evidence-based interventions require equipment, training, and time that may not be available in all settings; (4) surveillance and feedback: without robust data systems, it is difficult to identify practice gaps and track improvement; and (5) safety culture: when learning systems prioritize adherence over adaptation, innovation is delayed.

### 7.5 The role of specialized teams as organizational mechanisms

Rather than viewing specialized vascular access teams as an isolated intervention, they should be understood as the organizational mechanism that enables the implementation of all other technical interventions. Teams provide the infrastructure for standardized training, competency assessment, protocol development, quality monitoring, and continuous improvement (20). They serve as the bridge between evidence generation and clinical adoption, creating the conditions under which technical interventions can be consistently and safely delivered (70). The establishment of such teams represents not merely an additional intervention but a fundamental reorganization of how vascular access care is structured and delivered.

### 7.6 Future directions

Future research should move beyond simply calling for more randomized controlled trials. Priority areas include (1) implementation science: studies that identify effective strategies for overcoming clinical inertia and translating evidence into routine practice; (2) multicenter registries: large-scale data collection that enables risk adjustment, benchmarking, and identification of best practices; (3) competency assessment: validated tools for assessing and maintaining procedural skills; (4) continuous monitoring technologies: evaluation of intelligent infusion monitoring systems in neonatal populations; (5) artificial intelligence: AI-assisted tip positioning, risk prediction, and decision support; and (6) patient-centered outcomes: including pain, neurodevelopmental impact, and family experience.

## 8 Conclusion

This narrative review synthesizes current evidence on PICC-related complications in neonates and their preventive strategies. Key findings indicate that complication rates range from 10% to 40%, with infection, thrombosis, mechanical failure, and cardiac tamponade being the most clinically significant. Evidence-based interventions, including ultrasound guidance, intracavitary electrocardiography, tissue adhesive securement, and bundle care strategies, have been associated with reductions in specific complications in observational and quasi-experimental studies. However, the quality of supporting evidence varies considerably: ultrasound and IC-ECG are supported by moderate-level evidence, while tissue adhesive, antimicrobial catheters, and specialized teams rely mainly on low-level evidence from uncontrolled studies. The review proposes that neonatal vascular access management should evolve from fragmented, single-intervention approaches toward integrated, system-based frameworks. The 7-Rights Framework offers a conceptual structure for organizing the full spectrum of vascular access activities into a coherent model of proactive, patient-centered care. However, the translation of evidence into practice requires addressing human factors, overcoming clinical inertia, and strengthening implementation science approaches. Future research should prioritize multicenter prospective studies, the development of robust risk prediction models, the evaluation of intelligent monitoring technologies, and implementation strategies that bridge the gap between evidence and routine clinical practice.

## References

[1] Westergaard B Classen V Walther-Larsen S. Peripherally inserted central catheters in infants and children—indications, techniques, complications and clinical recommendations. Acta Anaesthesiol Scand. (2013) 57(3):278–87. 10.1111/aas.12024 23252685

[2] Ainsworth SB McGuire W. Peripherally inserted central catheters vs peripheral cannulas for delivering parenteral nutrition in neonates. JAMA. (2016) 315(23):2612–3. 10.1001/jama.2016.7020 27327804

[3] van Rens MR Van Der Lee R Spencer TR Van Boxtel T Barone G Crocoli A. The NAVIGATE project: a GloVANet-WoCoVA position statement on the nomenclature for vascular access devices. J Vasc Access. (2025) 26(5):1439–46. 10.1177/11297298241291248 39446461

[4] Shaw JCL. Parenteral nutrition in the management of sick low birthweight infants. Pediatr Clin N Am. (1973) 20(2):333–58. 10.1016/S0031-3955(16)32847-4

[5] Ozkiraz S Gokmen Z Ince DA Akcan AB Kilicdag H Ozel D. Peripherally inserted central venous catheters in critically ill premature neonates. J Vasc Access. (2013) 14(4):320–4. 10.5301/jva.5000157 23817952

[6] Bahoush G Salajegheh P Anari AM Eshghi A Aski BH. A review of peripherally inserted central catheters and various types of vascular access in very small children and pediatric patients and their potential complications. J Med Life. (2021) 14(3):298–309. 10.25122/jml-2020-0011 34377194 PMC8321608

[7] Avsar H Bulbul A Bas EK Uslu HS Unal ET. Peripherally inserted central catheters in newborns: a seven-year single-center experience from a neonatal intensive care unit. Children (Basel). (2025) 12(9):1168. 10.3390/children12091168 41007033 PMC12468454

[8] Xu YP Shang ZR Dorazio RM. Risk factors for peripherally inserted central catheterization-associated bloodstream infection in neonates. Zhongguo Dang Dai Er Ke Za Zhi. (2022) 24(2):141–6. 10.7499/j.issn.1008-8830.2109147 35209978 PMC8884050

[9] Zareef R Anka M Hatab T El Rassi I Yunis K Bitar F. Tamponade and massive pleural effusions secondary to peripherally inserted central catheter in neonates-A complication to be aware of. Front Cardiovasc Med. (2023) 10:1092814. 10.3389/fcvm.2023.1092814 36873398 PMC9981636

[10] Barone G Pittiruti M Biasucci DG Elisei D Iacobone E La Greca A. Neo-ECHOTIP: a structured protocol for ultrasound-based tip navigation and tip location during placement of central venous access devices in neonates. J Vasc Access. (2022) 23(5):679–88. 10.1177/11297298211007703 33818191

[11] Al Hamod DA Zeidan S Al Bizri A Baaklini G Nassif Y. Ultrasound-guided central line insertion and standard peripherally inserted catheter placement in preterm infants: comparing results from prospective study in a single-center. N Am J Med Sci. (2016) 8(5):205–9. 10.4103/1947-2714.183011 27298814 PMC4899959

[12] Zhang L Wang M Zhao M Pu S Zhao J Zhu G. Efficacy and safety of intracavitary electrocardiography-guided peripherally inserted central catheters in pediatric patients: a systematic review and meta-analysis. PeerJ. (2024) 12:e18274. 10.7717/peerj.18274 39399428 PMC11468838

[13] Gan W Hu L Luo Y Tang M. Impact of peripherally inserted central venous catheter-associated phlebitis in neonate guided by intracavitary electrocardiogram: a systematic review and meta-analysis of randomised controlled trials. Int Wound J. (2023) 20(4):1130–8. 10.1111/iwj.13971 36220149 PMC10031215

[14] Gilbert R Brown M Rainford N Donohue C Fraser C Sinha A. Antimicrobial-impregnated central venous catheters for prevention of neonatal bloodstream infection (PREVAIL): an open-label, parallel-group, pragmatic, randomised controlled trial. Lancet Child Adolesc Health. (2019) 3(6):381–90. 10.1016/S2352-4642(19)30114-2 31040096

[15] Bayoumi MAA Van Rens MFPT Chandra P Masry A D’Souza S Khalil AM. Does the antimicrobial-impregnated peripherally inserted central catheter decrease the CLABSI rate in neonates? Results from a retrospective cohort study. Front Pediatr. (2022) 10:1012800. 10.3389/fped.2022.1012800 36507144 PMC9730802

[16] de Souza S Takashima M Silva TL Nugyen L Kleidon TM Jardine L. Use of tissue adhesive for neonatal intravenous access devices: a scoping review. Eur J Pediatr. (2024) 183(12):5103–12. 10.1007/s00431-024-05800-3 39367917 PMC11527952

[17] Wang W Zhao C Ji Q Liu Y Shen G Wei L. Prevention of peripherally inserted central line-associated blood stream infections in very low-birth-weight infants by using a central line bundle guideline with a standard checklist: a case control study. BMC Pediatr. (2015) 15:69. 10.1186/s12887-015-0383-y 26084807 PMC4470111

[18] Zhu P Xu X Zhang X He X Jiao J. Effectiveness of evidence-based continuous quality improvement in reducing peripherally inserted central catheter complications among premature infants in China: a four-year retrospective study. J Multidiscip Healthc. (2025) 18:8153–67. 10.2147/JMDH.S552393 41459569 PMC12739944

[19] Van Rens M Ostroff M Bayoumi M. The modern role of neonatal PICCs subspecialty. Nurs Crit Care. (2025) 30(4):e70111. 10.1111/nicc.70111 40611458 PMC12231926

[20] Bayoumi MAA Van Rens MFP Chandra P Francia ALV D’Souza S George M. Effect of implementing an epicutaneo-caval catheter team in neonatal intensive care unit. J Vasc Access. (2021) 22(2):243–53. 10.1177/1129729820928182 32602399 PMC7983328

[21] van Rens MFPT Hugill K Van Der Lee R Piersigilli F Schwaberger B Mader S. Enhancing neonatal vascular access: proposing a patient-centered framework based on 7-rights. Pediatr Res. (2025). 10.1038/s41390-025-04521-z 41125962

[22] Li R Cao X Shi T Xiong L. Application of peripherally inserted central catheters in critically ill newborns experience from a neonatal intensive care unit. Medicine (Baltimore). (2019) 98(32):e15837. 10.1097/MD.0000000000015837 31393341 PMC6709114

[23] Ren X-L Li H-L Liu J Chen Y-J Wang M Qiu R-X. Ultrasound to localize the peripherally inserted central catheter tip position in newborn infants. Am J Perinatol. (2021) 38(2):122–5. 10.1055/s-0039-1694760 31412404

[24] Pallickal AM Pappa O. In neonates with a peripherally inserted central catheter (n-PICC), does limb position influence catheter tip location on x-ray? Arch Dis Child. (2026) 111:457–9. 10.1136/archdischild-2025-329603 41475894

[25] Nadroo AM Glass RB Lin J Green RS Holzman IR. Changes in upper extremity position cause migration of peripherally inserted central catheters in neonates. Pediatrics. (2002) 110(1 Pt 1):131–6. 10.1542/peds.110.1.131 12093958

[26] Connolly B Amaral J Walsh S Temple M Chait P Stephens D. Influence of arm movement on central tip location of peripherally inserted central catheters (PICCs). Pediatr Radiol. (2006) 36(8):845–50. 10.1007/s00247-006-0172-8 16758187

[27] Paulson PR Miller KM. Neonatal peripherally inserted central catheters: recommendations for prevention of insertion and postinsertion complications. Neonatal Netw. (2008) 27(4):245–57. 10.1891/0730-0832.27.4.245 18697655

[28] Chen X Zhou L Tan Y. Selection of PICC catheter location in neonates via evidence-based ACE star model. Zhong Nan Da Xue Xue Bao Yi Xue Ban. (2020) 45(9):1082–8. 10.11817/j.issn.1672-7347.2020.190613 33051422

[29] Naik VM Mantha S Rayani BK. Vascular access in children. Indian J Anaesth. (2019) 63(9):737–45. 10.4103/ija.IJA_489_19 31571687 PMC6761776

[30] D'Andrea V Prontera G Pinna G Cota F Fattore S Costa S. Securement of umbilical venous catheter using cyanoacrylate glue: a randomized controlled trial. J Pediatr. (2023) 260:113517. 10.1016/j.jpeds.2023.113517 37244573

[31] Bradford NK Edwards RM Chan RJ. Normal saline (0.9% sodium chloride) versus heparin intermittent flushing for the prevention of occlusion in long-term central venous catheters in infants and children. Cochrane Database Syst Rev. (2020) 4(4):D10996. 10.1002/14651858.CD010996.pub3 PMC7192095 32352563

[32] Nascimento AP De Medeiros KS Costa APF Sarmento AC Cruz GKP Gonçalves AK. Heparin versus 0.9% sodium chloride intermittent flushing for preventing occlusion in newborns with peripherally inserted central catheters: a systematic review protocol. PLoS One. (2022) 17(12):e278068. 10.1371/journal.pone.0278068 PMC9803159 36584103

[33] van Rens MFPT Hugill K Van Der Lee R Francia ALV Van Loon FHJ Bayoumi MAA. Comparing conventional and modified seldinger techniques using a micro-insertion kit for PICC placement in neonates: a retrospective cohort study. Front Pediatr. (2024) 12:1395395. 10.3389/fped.2024.1395395 38756973 PMC11096449

[34] Keir A Giesinger R Dunn M. How long should umbilical venous catheters remain in place in neonates who require long-term (≥5–7 days) central venous access? J Paediatr Child Health. (2014) 50(8):649–52. 10.1111/jpc.12690 25080979

[35] Butler-O’Hara M Buzzard CJ Reubens L McDermott MP DiGrazio W D’Angio CT. A randomized trial comparing long-term and short-term use of umbilical venous catheters in premature infants with birth weights of less than 1251 grams. Pediatrics. (2006) 118(1):e25–35. 10.1542/peds.2005-1880 16785289

[36] Hess S Poryo M Böttger R Franz A Klotz D Linnemann K. Umbilical venous catheter- and peripherally inserted central catheter-associated complications in preterm infants with birth weight <1250 g: results from a survey in Austria and Germany. Wien Med Wochenschr. (2023) 173(7–8):161–7. 10.1007/s10354-022-00952-z 35939216 PMC10147741

[37] Kisa P Ting J Callejas A Osiovich H Butterworth SA. Major thrombotic complications with lower limb PICCs in surgical neonates. J Pediatr Surg. (2015) 50(5):786–9. 10.1016/j.jpedsurg.2015.02.043 25783362

[38] Hu Y Ling Y Ye Y Zhang L Xia X Jiang Q. Analysis of risk factors of PICC-related bloodstream infection in newborns: implications for nursing care. Eur J Med Res. (2021) 26(1):80. 10.1186/s40001-021-00546-2 34301331 PMC8299687

[39] Zhang Y Li S Li Y Zheng J Dong Y. Analysis and risk factors of deep vein catheterization-related bloodstream infections in neonates. Medicine (Baltimore). (2024) 103(12):e37184. 10.1097/MD.0000000000037184 38518044 PMC10956940

[40] Sharpe EL Curry S Wyckoff MM. NANN Neonatal peripherally inserted central catheters: guideline for practice, 4th ed. Adv Neonatal Care. (2024) 24(4):313–5. 10.1097/ANC.0000000000001182 39052577

[41] Bayoumi MAA Van Rens R Chandra P Shaltout D Gad A Elmalik EE. Peripherally inserted central catheters versus non-tunnelled ultrasound-guided central venous catheters in newborns: a retrospective observational study. BMJ Open. (2022) 12(4):e58866. 10.1136/bmjopen-2021-058866 PMC8987782 35387831

[42] O'Grady NP Alexander M Burns LA Dellinger EP Garland J Heard SO. Guidelines for the prevention of intravascular catheter-related infections. Clin Infect Dis. (2011) 52(9):e162–93. 10.1093/cid/cir257 21460264 PMC3106269

[43] Wang CN Deng HR. Percutaneous endovenous intervention plus anticoagulation versus anticoagulation alone for treating patients with proximal deep vein thrombosis: a meta-analysis and systematic review. Ann Vasc Surg. (2018) 49:39–48. 10.1016/j.avsg.2017.09.027 29454036

[44] van Rens MFPT Paramban R Francia ALV Chandra P Mahmah MA Thome UH. Evaluation of a diluted lipid emulsion solution as a lubricant for improved peripherally inserted central catheter guidewire removal in a neonatal population. BMC Pediatr. (2022) 22(1):71. 10.1186/s12887-022-03119-2 35094692 PMC8802504

[45] Pettit J. Technological advances for PICC placement and management. Adv Neonatal Care. (2007) 7(3):122–31. 10.1097/01.ANC.0000278210.18639.fd 17844776

[46] Jain A Deshpande P Shah P. Peripherally inserted central catheter tip position and risk of associated complications in neonates. J Perinatol. (2013) 33(4):307–12. 10.1038/jp.2012.112 22955288

[47] Gavin NC Webster J Chan RJ Rickard CM. Frequency of dressing changes for central venous access devices on catheter-related infections. Cochrane Database Syst Rev. (2016) 2(2):CD009213. 10.1002/14651858.CD009213.pub2 PMC8765739 26827714

[48] van Rens MFPT Bayoumi MAA Van De Hoogen A Francia ALV Cabanillas IJ Van Loon FHJ. The ABBA project (assess better before access): a retrospective cohort study of neonatal intravascular device outcomes. Front Pediatr. (2022) 10:980725. 10.3389/fped.2022.980725 36405839 PMC9670536

[49] Costa P Kimura AF Brandon DH Paiva ED Camargo PP. The development of a risk score for unplanned removal of peripherally inserted central catheter in newborns. Rev Lat Am Enfermagem. (2015) 23(3):475–82. 10.1590/0104-1169.0491.2578 26155011 PMC4547071

[50] Duwadi S Zhao Q Budal BS. Peripherally inserted central catheters in critically ill patients—complications and its prevention: a review. Int J Nurs Sci. (2019) 6(1):99–105. 10.1016/j.ijnss.2018.12.007 31406874 PMC6608659

[51] Yu X Yue S Wang M Cao C Liao Z Ding Y. Risk factors related to peripherally inserted central venous catheter nonselective removal in neonates. Biomed Res Int. (2018) 2018:3769376. 10.1155/2018/3769376 30003096 PMC5998161

[52] Jones AM Mongan S Ullman A August D Sharpe E Alderman AA. Umbilical venous catheter and peripherally inserted central catheter malposition and tip migration in neonates: a mixed methods cost analysis. Int J Nurs Stud Adv. (2025) 9:100450. 10.1016/j.ijnsa.2025.100450 41322982 PMC12664355

[53] Hou A Fu J. Pericardial effusion/cardiac tamponade induced by peripherally inserted central catheters in very low birth weight infants: a case report and literature review. Front Pediatr. (2020) 8:235. 10.3389/fped.2020.00235 32500049 PMC7242726

[54] Igarashi A Okuno T Shimizu T Ohta G Ohshima Y. Mechanical stimulation is a risk factor for phlebitis associated with peripherally inserted central venous catheter in neonates. Pediatr Int. (2021) 63(5):561–4. 10.1111/ped.14476 32964580

[55] Yilmaz FH Çeri A. A worrisome PICC line complication in a premature newborn: knotted catheter. Indian J Pediatr. (2021) 88(9):939. 10.1007/s12098-021-03820-7 34106440

[56] He L Ye Q Huang L Wang M He M Li B. Using phlebotomy to remove a difficult peripherally inserted central catheter insertion and removal in very low birth weight infants: case report of a rare complication. AME Case Rep. (2024) 8:51. 10.21037/acr-23-145 38711880 PMC11071004

[57] Trinh HT Nguyen TT Nguyen TT. Cardiac tamponade due to pericardial effusion following peripherally inserted central catheter: a single-institution case series. Cureus. (2024) 16(3):e56403. 10.7759/cureus.56403 38638757 PMC11025877

[58] Gupta R Drendel A Hoffmann R Quijano C Uhing M. Migration of central venous catheters in neonates: a radiographic assessment. Am J Perinatol. (2016) 33(6):600–4. 10.1055/s-0035-1570341 26731179

[59] Escobar Gimenes FR Menegueti MG Nunes E Silva RP Marques AR Santos VB. Ultrasound-Guided peripherally inserted central catheter insertion: a nurse training initiative. J Radiol Nurs. (2025) 44(2):210–4. 10.1016/j.jradnu.2024.12.003

[60] Liu X Tao X Xu Y Zhang X Chen Y Wu L. Comparison of bedside ultrasonography and bedside chest radiography in neonatal peripherally inserted central catheters: a before and after self-control study. Front Pediatr. (2022) 10:976826. 10.3389/fped.2022.976826 36330366 PMC9623023

[61] Hall S August D Hall J Ullman AJ Marsh N. Insertion technologies for neonatal venous access: a scoping review. J Perinatol. (2026). 10.1038/s41372-026-02661-6 41998176

[62] Xiong L Tan Y Yang X Wang H Ding M Sessler DI. Catheter-related internal jugular vein thrombosis in neonates and long-term consequences: a prospective cohort study. Anesthesiology. (2025) 142(2):298–307. 10.1097/ALN.0000000000005250 39365956

[63] Rossetti F Pittiruti M Lamperti M Graziano U Celentano D Capozzoli G. The intracavitary ECG method for positioning the tip of central venous access devices in pediatric patients: results of an Italian multicenter study. J Vasc Access. (2015) 16(2):137–43. 10.5301/jva.5000281 25198817

[64] Zhou M Zeng W-L Lu CD Sun M-W Jiang H. Intracavitary electrocardiogram guidance for peripherally inserted central catheter placement: a systematic review and trial sequential meta-analysis. J Vasc Access. (2026) 27(1):227–38. 10.1177/11297298251334889 40312860

[65] Regne GRS Galvão Diniz C Manzo BF. Strategies to ensure central PICC line positioning in neonates: scoping review. J Neonatal Nurs. (2025) 31(5):101716. 10.1016/j.jnn.2025.101716

[66] van Rens M Nimeri AMA Spencer TR Hugill K Francia ALV Olukade TO. Cyanoacrylate securement in neonatal PICC use: a 4-year observational study. Adv Neonatal Care. (2022) 22(3):270–9. 10.1097/ANC.0000000000000963 34743117

[67] Pronovost P Needham D Berenholtz S Sinopoli D Chu H Cosgrove S. An intervention to decrease catheter-related bloodstream infections in the ICU. N Engl J Med. (2006) 355(26):2725–32. 10.1056/NEJMoa061115 17192537

[68] Krysiak K Cleary B McCallion N O’Brien F. The effect of patient’s body weight, infusion connection point, and infusion pump position on intravenous multi-infusion drug delivery at low infusion rates suitable for premature neonates. J Pharm Pharmacol. (2024) 76(1):34–43. 10.1093/jpp/rgad108 38041860

[69] van Rens MFPT Hugill K Van Der Lee R Francia ALV Van Loon FHJ Bayoumi MAA. Evaluation of optical sensor technology for the early detection of peripheral intravenous infiltration in neonates: a retrospective cohort study. BMJ Open. (2025) 15(7):e94464. 10.1136/bmjopen-2024-094464 PMC12228456 40617616

[70] Legemaat MM Jongerden IP Van Rens RMFPT Zielman M Van Den Hoogen A. Effect of a vascular access team on central line-associated bloodstream infections in infants admitted to a neonatal intensive care unit: a systematic review. Int J Nurs Stud. (2015) 52(5):1003–10. 10.1016/j.ijnurstu.2014.11.010 25526669

[71] Bayoumi MAA Elmalik EE Ali H D’Souza S Furigay J Romo A. Neonatal simulation program: a 5 years educational journey from Qatar. Front Pediatr. (2022) 10:843147. 10.3389/fped.2022.843147 35386259 PMC8977624

[72] Krein SL Kuhn L Ratz D Chopra V. Use of designated nurse PICC teams and CLABSI prevention practices among U.S. Hospitals: a survey-based study. J Patient Saf. (2019) 15(4):293–5. 10.1097/PTS.0000000000000246 26558650

[73] Comber ER August D Nguyen LN De Souza S Judith Ullman A Byrnes J. Implementation frameworks, strategies, and outcomes in optimizing central venous access device practice in neonates: a scoping review. Hosp Pediatr. (2025) 15(8):e404–15. 10.1542/hpeds.2024-008248 40690977

[74] Van Rens M Hugill K Bayoumi M. When success becomes a barrier: tackling inertia in necessary change in clinical practice in neonatal vascular access. BMJ Paediatr Open. (2026) 10(1):e004848. 10.1136/bmjpo-2026-004848 42167901 PMC13202137

